# An extensive study of potential inhibitors of extracellular vesicles release in triple-negative breast cancer

**DOI:** 10.1186/s12885-023-11160-2

**Published:** 2023-07-13

**Authors:** Niamh McNamee, Mariadelva Catalano, Anindya Mukhopadhya, Lorraine O’Driscoll

**Affiliations:** 1 School of Pharmacy and Pharmaceutical Sciences, Trinity College Dublin, Dublin, Ireland; 2 Trinity Biomedical Sciences Institute, Trinity College Dublin, Dublin, Ireland; 3 Trinity St. James’s Cancer Institute, Trinity College Dublin, Dublin, Ireland

**Keywords:** Extracellular vesicles, Inhibition, Triple-negative breast cancer

## Abstract

**Background:**

Cancer cells release heterogeneous populations of extracellular vesicles (EVs) that transmit aggressive phenotypic traits to recipient cells. We aimed to establish if the heterogenous EVs population or a sub-population is responsible, if we could block undesirable cell-to-cell communication by EVs, and, if some EVs continued to be released, would their undesirable influences on recipient cells continue.

**Methods:**

Three triple-negative breast cancer (TNBC) cell lines were used. Non-toxic concentrations of calpeptin, Y27632, manumycin A, GW4869 and combinations thereof were tested to block EVs. Ultracentrifugation-based methods collected EVs, which were then characterised by nanoparticle tracking analysis, immunoblotting, and transmission electron microscopy. A quick screening flow cytometry method evaluated EVs in solution. The influences of EVs on recipient cells’ migration was investigated.

**Results:**

All EV sub-populations were apparently involved in transmitting undesirable phenotypic characteristics. All compounds/combinations significantly (64–98%) reduced EVs’ release. Our quick screening broadly reflected our more comprehensive EVs analysis. The 2–36% of EVs that continued to be released caused less transmission to recipient cells, but not on a comparable scale to the reduction of EVs release achieved.

**Conclusion:**

Up to 98% inhibition of EVs’ release was achieved. To prevent the transmission of undesirable phenotypic traits by EVs, their total inhibition may be necessary.

**Supplementary Information:**

The online version contains supplementary material available at 10.1186/s12885-023-11160-2.

## Background

Extracellular vesicles (EVs) are membrane-enclosed nano-sized sacs that are released by most, if not all, cells in the body. EVs are involved in cell-to-cell communication. Ourselves and others have reported tumour-derived EVs to transfer their cargo to recipient cells, changing the profile and tumorigenic potential, increasing migration, invasion and metastasis of the cancer cells as well as their resistance to chemotherapy drugs [1]. The collective term EVs includes both microvesicles and exosomes and is heterogenous in nature encompassing small, medium, and large EVs sub-populations [2]. However, when EVs are released into the extracellular environment from their cell of origin, it is not possible to be certain if they have been formed through an endosomal pathway or by budding from the membrane based on their size alone. This can be somewhat informed by the markers that are carried by the EVs. Larger EVs such as microvesicles that bud from the cell membrane -and so are often referred to as ectosomes- are claimed to contain markers such as ARF6 (i.e. a protein involved in cytoskeleton remodelling) [3], actinin-4, mitofilin [4] and cytokeratin 16 [5]. Exosomes have been reported to carry proteins involved in the endosomal pathways, such tumour susceptibility gene 101 (TSG101), a protein which makes up part of the ESCRT1 complex, recognising and sorting cargo into intraluminal vesicles [6]. Syntenin, an adaptor protein, syndecans and ALIX are other proteins that may be carried in the exosomes sub-group of EVs [7]. However, it must be considered that there are no definitive exosomal or definitive ectosomal markers reported to date. Also, very small EVs can also bud from the cell membrane; thus, they are the typical size of exosomes, but are actually ectosomes.

Triple-negative breast cancer (TNBC) is an aggressive cancer, often associated with younger women and leading to a poor prognosis. Although it accounts for only 15–20% of breast cancers, it is responsible for a disproportionate number of deaths. Several studies of TNBC have demonstrated negative effects by released EVs. In what we understand to be the first such study, our group reported EVs involvement in propagating the disease by demonstrating that EVs from the very “aggressive” TNBC cell line variant, Hs578Ts(i)_8_ [8] could transfer its aggressive phenotypic traits to recipient cells, reflecting the phenotype of the cells of EV origin. For example, Hs578Ts(i)_8_ EVs, when taken up by the isogenic but less aggressive Hs578T cells, caused increased proliferation, migration, and invasion, as well as including neovascularisation and angiogenesis [9, 10]. Since that study, other studies have shown that EVs from the TNBC cell line MDA-MB-231 transfer adhesion proteins to recipient breast cancer cells, MDA-MB-231 and T47D, increasing the migration and invasion of those cells [11].

Together this suggested to us that to prevent many of the serious issues associated with cancer, if a tumour cannot be completely removed, blocking EVs release from the cancer cells could be a relevant therapeutic option; whether that involved blocking heterogeneous population of EVs, or a specific sub-population of EVs, being released from the tumour cells and that was deemed responsible for these problems. Regarding potential inhibitors, we previously reviewed the literature [12] and, while there are many suggested inhibitors, often these compounds were previously evaluated at concentrations that would be toxic to cells, meaning that their specific influence on EVs release would be very difficult, if at all possible, to address due to the overall destruction of the cells and so release of substantial cellular debris. Furthermore, these studies typically did not adhere to MISEV2018 guidelines which are the minimal information suggested for studies of EVs that have been agreed upon by > 350 EVs researchers from around the world [13]. It also must be noted that to date there has been no extensive study on blocking EVs release which includes and compares a number of potential inhibitors, as well as being considerate of MISEV2018 guidelines.

**Fig. 1:**
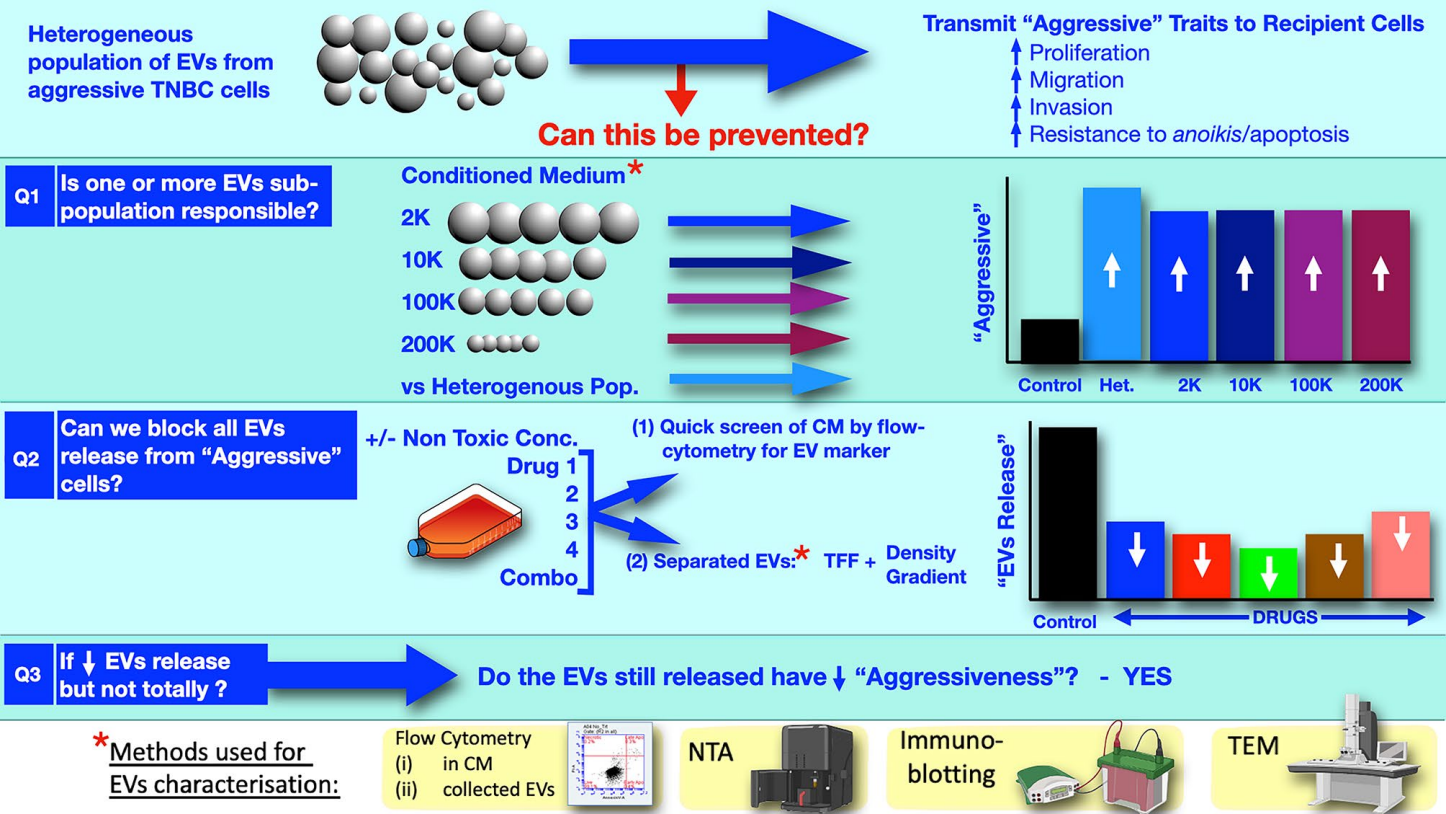
Study design. Considering that the heterogenous population of EVs from TNBC cells can transmit a broad range of undesirable phenotypic traits to recipient cancer and normal cells, this study investigated if the heterogenous EVs population or a sub-population is responsible; if we could block undesirable cell-to-cell communication by EVs; and, if some EVs continued to be released, would their undesirable influences on recipient cells continue. EVs were collected using ultracentrifugation-based method and extensively characterised. An imaging flow cytometry method for assessing EVs in solution was compared to the more comprehensive analysis of collected EVs. Influences of EVs on recipient cells were evaluated

Thus, advancing on the discovery that the heterogeneous pool of EVs from the very aggressive TNBC cells can transmit their undesirable phenotypic traits to other cancer and normal cell types [9, 10], here we sought to first establish if a particular sub-population of EVs, rather than the heterogeneous EVs population, might be causally involved and so targetable. We then wished to undertake a comprehensive evaluation of non-toxic concentrations of four potential EV inhibitors i.e., calpeptin, Y27632, manumycin A and GW4869 on the release of the EVs (whether that be the heterogenous EVs population or a specific sub-population of EVs, informed by our initial studies) from the highly aggressive Hs578Ts(i)_8_ cell line. Finally, if total blocking of EVs release from the aggressive cells was not achievable, we were interested to determine if the effects, on recipient cells, of the EVs that continued to be released -after efforts to block their release- would be modified in anyway (Fig. 1).

## Methods

### Cell culture and drugs/compounds

Three triple-negative breast cancer (TNBC) cell line variants were cultured based on the recommendation of the American Tissue Culture Collection (ATCC). Hs578T cell line and its isogenic sub-clone Hs578Ts(i)_8_ were cultured in DMEM (Sigma-Aldrich,Cat.#:D5671) containing 10% foetal bovine serum (FBS) (Gibco, Cat.#:10,270,106), 2mM L-Glutamine (Sigma-Aldrich, Cat.#:10,516) and 10 µg/ml insulin (Sigma-Aldrich, Cat.#:19,278). BT549 cells were cultured in RPMI medium (Sigma-Aldrich, Cat.#:R0883) containing 10% FBS and 2mM L-Glutamine. For experiments that included EVs collection, EVs testing, etc., completed FBS in media was replaced with EVs-depleted FBS (i.e. dFBS) in media. For this, FBS was depleted of EVs by overnight ultracentrifugation at 120,000 g at 10 °C. After ultracentrifugation, approximately 15mL of dFBS from the top (i.e. clear liquid which still contains EV-sized particles) was removed with the rest being collected, leaving approximately 2mL at the bottom as to not disturb the pellet. All cell lines were cultured at 37 °C, 5% CO_2_. *Mycoplasma* testing was routinely performed by reverse-transcriptase polymerase chain reaction (ATCC, Cat.#:30-1012 K).

Compounds tested were calpeptin (Apexbio, Cat.#:A4411), Y27632 (Apexbio, Cat.#:A3008), GW4869 (Selleckchem, Cat.#:S7609) and manumycin A (Sigma, Cat.#:M6418).

### Toxicity of compounds being evaluated

In order to select suitable drug concentrations that were non-toxic to the cells, so as to establish if they truly influence EVs release, effects of the drugs on the cells were evaluated by acid phosphatase assay and confirmed by flow cytometry.

#### Acid phosphatase assay

Hs578Ts(i)_8_ cells were seeded at 3 × 10^3^ per well of a 96-well plate and allowed to attach overnight. Cells were treated with the drugs/compounds (listed above) the next day and cultured for 48 h. The acid phosphatase assay was performed as previously published to determine the cell viability [14].

#### Flow cytometry assay

Toxicity of the compounds was also investigated using annexin V-FITC (IQ Products, Cat.#:IQP-120 F) and propidium iodide (PI) staining (BD Biosciences, Cat.#:550,474). Hs578Ts(i)_8_ cells were seeded at 5 × 10^4^ per well in 6-well plate. The next day the cells were treated with drugs at the concentration selected by acid phosphatase assay. 48 h later the conditioned medium (CM) was collected. The cells were trypsinised and placed with the CM and centrifuged at 1200 rpm for 5 min. The pellet was resuspended in 500 µl of 1X Binding Buffer (BB) (for 20X BB: 2.6 g of HEPES (10.9mM), 8.18 g of NaCl (140 mM), 0.28 g of CaCl_2_ (2.5 mM), pH 7.4) and centrifuged at 600 g for 5 min. Annexin V-FITC (diluted 1:33.3 with BB) was used to resuspend the pellet and incubated on ice for 20 min. 500 µl of BB was added and centrifuged at 600 g for 5 min at 4 °C. The pellet was resuspended in 500 µl of BB and 5 µl of PI staining solution. Apoptosis was analysed on 1 × 10^4^ events using a BD Accuri™ C6 flow cytometer according to published protocol [15].

### Collection of conditioned medium and imaging flow cytometry as a “quick screen” of changes in quantities of EVs released

We wished to establish if we could develop a quick screen method to get an initial indication of EVs release and changes by not separating the EVs from the CM but, instead, screening the medium -which had been cleared of cells- directly for the presence of an EV marker, CD9.

For this, Hs578Ts(i)_8_ cells were seeded in 24-well plates in 10% FBS-containing medium, at 1.5 × 10^4^ per well. After 24 h, the medium was removed and replaced with 500 µl of medium containing 10% dFBS, with or without the compounds being tested. After 48 h, the CM was collected, the cells were counted, and viability was measured. The CM was then cleared of debris by centrifuging at 300 g for 5 min at 4 °C and was screened for the presence of CD9. For this, the anti-CD9 antibody (ExBio, Cat.#:1P-208-T100) was diluted in PBS (1:25) and centrifuged at 16,000 g for 15 min at 4 °C to remove antibody aggregates. The CM was incubated with the anti-CD9 antibody (1:1) for 2 h at room temperature in the dark. After incubation, the CM-antibody sample was washed using a 300 kDa Nanosep centrifugal filter (Nanosep, Cat.#:516–8531), centrifuging at 2,500 g for 3 min to remove any unbound antibody. The cleaned CM-antibody sample was resuspended in PBS and was analysed within 2 h on an ImageStream X MK II imaging flow cytometer at 60X magnification and low flow rate. PBS and unstained controls were run in parallel. All samples were screened for 5 min. Once screened, 4% of NP-40 (EMD Millipore, Cat.#:492,016) was added at 1:1 ratio with the sample and was re-screened as the NP-40 control. Data analysis was performed using IDEAS software v6.2. EVs in the CM were detected based on brightfield detection and SSC visibility [16]. Large EVs (lEVs) were gated events with clear brightfield and SSC signals, medium EVs (mEVs) events with low/no brightfield and lower but clearly visible SSC signals, while small EVs (sEVs) have no visible brightfield and no/very low SSC signals. All three sub-populations of EVs were combined when calculating the total heterogeneous EVs numbers present in each sample. This protocol was adapted from a previously published protocol [16].

### Extracellular vesicles collection

#### Heterogeneous EV

The heterogeneous population of EVs were collected as we previously described [9].

#### EVs sub-populations

Hs578Ts(i)_8_ cells were seeded at 5 × 10^5^ cells per T175 flask. The following day the medium was replaced with DMEM medium supplemented with 10% dFBS and 1% penicillin/streptomycin and the cells were cultured for another 5 days. EVs were collected by differential ultracentrifugation, adapted from a previously published protocol [4]. Specifically, CM was collected and the cell number was counted. The CM was centrifuged at 300 g for 10 min at 4 °C, three times, as a pre-clearing step to remove cellular debris. The CM was transferred to new 50ml tubes and centrifuged at 2,000 g for 20 min at 4 °C (producing the 2 K pellet). The CM was transferred to Quickseal 39ml tubes (Beckman coulter, Cat. #:342,414) and centrifuged in a Type 70 Ti fixed angle rotor (Beckman coulter, Cat. #:337,922) at 10,000 g for 30 min (producing the 10 K pellet). The CM was transferred to new Quickseal tube and centrifuged in a Type 70 Ti rotor at 100,000 g for 70 min (producing the 100 K pellet). Finally, the CM was transferred to another Quickseal tube and centrifuged in a 70 Ti rotor at 200,000 g for 65 min (producing the 200 K pellet). All pellets were washed with PBS and re-centrifuged at the same speed as previously, before resuspending each EVs sub-population pellet in 150 µl of PBS and storing in Protein LoBind tubes (Eppendorf, Cat. #: 0030 108.116) at -80 °C.

#### EVs collected after inhibitor treatment

Hs578Ts(i)_8_ cells were seeded at 1.5 × 10^6^ cells per T175 flask. The following day the medium was replaced with DMEM supplemented with 10% dFBS and 1% penicillin/streptomycin and cultured for another 48 h in the presence of the proposed EVs inhibitors at a chosen concentration. CM was centrifuged at 300 g for 10 min to remove cellular debris. This spin was repeated three times in total. The CM was concentrated from approximately 125mL to 1.5mL using a tangential flow filtration (HansaBioMed, Cat.#:HBM-TFF/1). The 1.5mL of concentrated CM was then loaded at the bottom of an Optiprep™ (Sigma, Cat.#:D1556) density gradient to separate EVs based on their density. In 17 mL Ultraclear UC tubes (Beckman Coulter, Cat.#:344,061), 1.5 mL of CM was diluted with 60% Optiprep and PBS to form the bottom layer of 40% (8 mL in total). 30% (2.5 mL), 20% (2.5 mL), 10% (2.5 mL) and 5% (2 mL) of Optiprep was loaded on top, in subsequent layers. The Optiprep gradient was ultracentrifuged at 186,000 g for 18.5 h at 4 °C in a SW 32.1 Ti swinging rotor (Beckman Coulter, Cat.#:369,651). Fractions of 1mL were collected from top to bottom. Fractions within the density range of 1.03 g/mL-1.19 g/mL (i.e. fractions 3–9) were pooled and ultracentrifuged in a Quickseal (39ml) tubes (Beckman coulter, Cat.#:342,414) in a Type 70 Ti fixed angle rotor (Beckman coulter, Cat.#:337,922) at 120,000 g for 2 h. This was repeated a second time and the final pellet was resuspended in 150 or 200 µl of sterile PBS, depending on the starting cell/flask numbers. These re-suspended EVs were stored in Protein LoBind tubes (Eppendorf, Cat.#:0030 108.116) at -80 °C.

### Extracellular vesicles characterisation

#### Protein quantification

A Bradford assay, using the Bio-Rad protein assay dye reagent (Bio-Rad, Cat.#:500-0006), was used to measure the protein concentration of the EVs isolates.

#### Nanoparticle tracking analysis

Nanoparticle tracking analysis (NTA), a method used to estimate particles/EVs concentration and size, was performed as we previously described [17].

#### Transmission electron microscopy (TEM)

10µL samples of EVs/isolates suspension were placed on formvar carbon-coated nickel grids (Ted Pella Inc, Cat.#:01813-F) and allowed settle for 10 min. A droplet of paraformaldehyde (2%) was placed on parafilm and the grid was placed on top and fixed for 10 min. This was then contrasted in 2% uranyl acetate (BDH, Cat.#:230,550) and all images were taken using a JEOL JEM-2100 TEM at 120 kV.

#### Immunoblotting

Immunoblotting was performed as we previously described [9], loading 10 µg of protein per lane of the gel. Primary antibodies which are anti-CD63 (1/500; Abcam, Cat.#:ab68418), anti-syntenin (1/1000; Abcam, Cat.#:ab133267), anti-TSG101 (1/1000; Abcam, Cat.#:ab83), anti-CD81 (1/250; Santa Cruz, Cat.#: 5A6), anti-GRP94 (1/2000; Cell Signalling, Cat.#:2104 S), and anti-calnexin (1/1000; Abcam, Cat.#:ab92573) were used. Secondary antibodies used were anti-mouse (1/1000 in 5% BSA/PBS-T, Cell Signalling, Cat.#:7076) or anti-rabbit (1/1000 in 5% BSA/PBS-T, Cell Signalling, Cat.#:7074). Lysate of Hs578Ts(i)_8_ cells were included on all gels as a control and densitometric analysis was performed using Fiji software. Of note: cropped images of blots are included in the main manuscript and the associated full blots can be found in the Supplemental Material.

### Cell migration assays

Cell migration assays were performed based on our previously published method [9]. Hs578T and BT549 cells were seeded at 4 × 10^4^ and 3 × 10^4^, respectively, per well in 24-well plates. These were allowed to develop into monolayers by incubating overnight. A 200µl pipette tip was then used to create a scratch down the centre of the monolayer. 500µl of medium including 1% dFBS and containing EVs was added to the wells. The wound closure/”healing” was monitored over 24 h by phase-contrast microscopy using an Olympus IX81 inverted microscope and analysed using ImageJ software.

### Statistical analysis

Statistical analysis of the data was performed using GraphPad Prism. Graphs were generated using GraphPad Prism 9. P-values were generated using Student’s t-test or ANOVA for multiple comparisons. P < 0.05 was considered statistically significant.

## Results

### Cytotoxicity of potential drugs/compounds

**Fig. 2:**
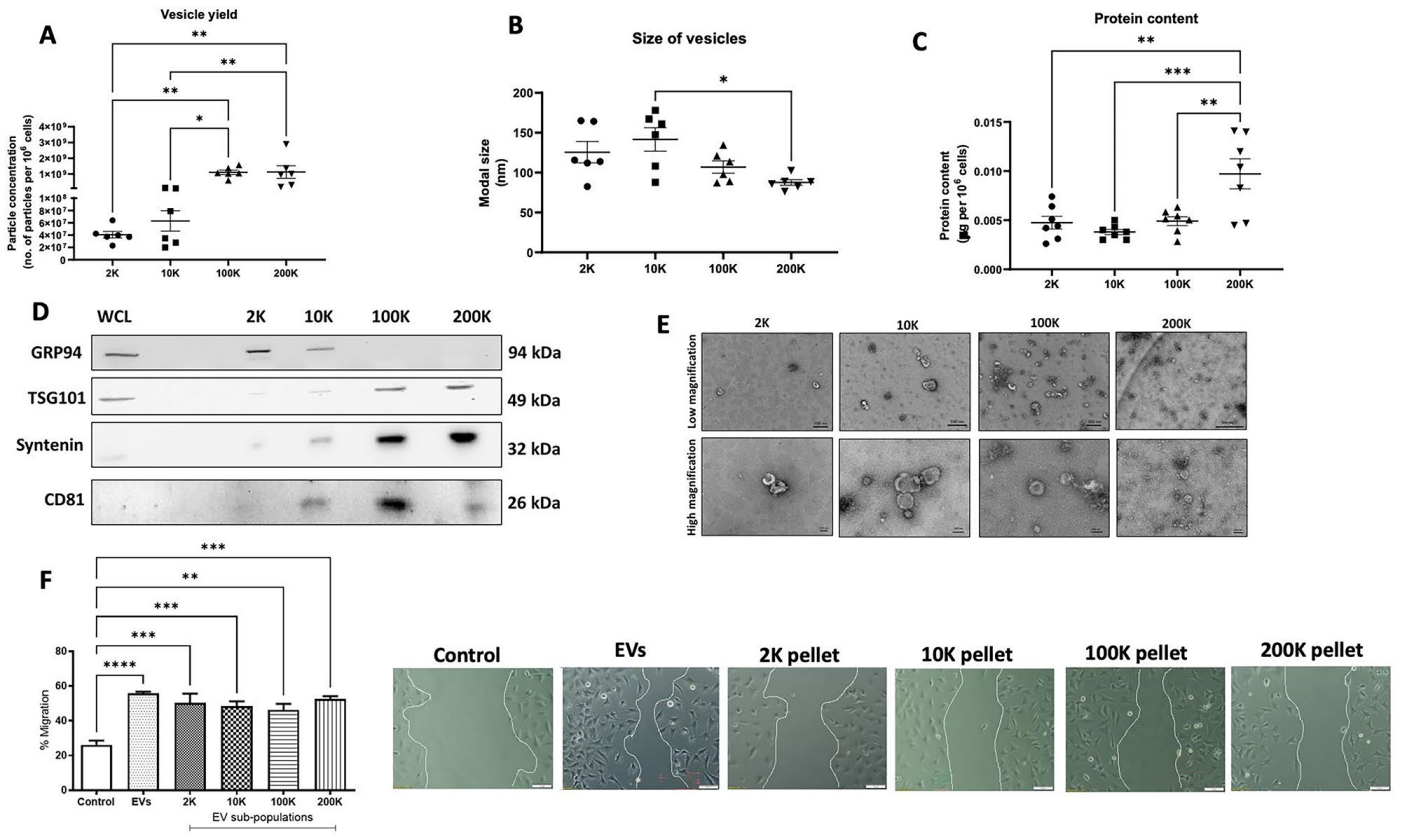
EV sub-populations (**A**) yield, (**B**) size were measured by NTA and (**C**) protein content by Bradford assay. Immunoblotting (**D**) was performed, with Hs578Ts(i)_8_ cell lysate used as control. WCL = Whole cell lysate. TEM analysis (**E**) was also performed. Hs578T cells were treated with Hs578Ts(i)_8_-derived heterogeneous EVs and EVs’ sub-populations and a migration assay was performed. Scale bars on TEM images are 500 nm (low magnification) and 100 nm (high magnification). Full-length blots/gels are presented in Supplementary Fig. 2. Graph (**F**) represents mean ± SEM of *n = 4* independent biological experiments. One-way ANOVA was used as a statistical test for all graphs. *P < 0.05, **P < 0.01, ***P < 0.001, ****P < 0.0001

Having previously discovered that the total heterogenous population of TNBC Hs578Ts(i)_8_ cells’ EVs transmit aggressive traits -representing their cells of origin- to more docile Hs578T cells [9], we investigated -using cell migration as representative phenotypic characteristic- if a particular EVs sub-population may be responsible for this effect or if the total heterogenous population likely worked together. Thus, four sub-populations of EVs were separated by differential ultracentrifugation, extensively characterised, and compared to the heterogeneous population (Fig. 2(A-E)). By increasing centrifugation speeds we aimed to separated large (2 K pellet), medium (10 K pellet), and small (100 and 200 K pellets) sub-populations [4]. NTA analysis indicated that the Hs578Ts(i)_8_ 100 K pellet contained significantly more EVs compared to the 2 K sub-population and compared to the 10 K sub-population (Fig. 2(A)). As expected, the 200 K sub-population presented the smallest particles, having significantly smaller sizes compared to the 10 K pellet (Fig. 2(B)). The 200 K sub-population also had significantly more protein compared to the other three sub-populations including 2 K, 10 K, and 100 K (Fig. 2(C)). This was unsurprising as it contained significantly more EVs. The TNBC EVs sub-populations were also characterised by immunoblot (Fig. 2(D)) and TEM (Fig. 2(E)). Once it was confirmed that EVs sub-populations were successfully collected, the influences of these EVs were tested on the parent, docile Hs578T cells’ migratory properties (Fig. 2 (F)). In brief, we confirmed that the heterogenous population of EVs does, indeed, significantly increase migration of the recipient cells. Interestingly, we discovered that each of the 4 EVs’ sub-populations also induced significant migration, but to similar extents to each other. Furthermore, each had slightly lower influence than the heterogeneous population of EVs. This suggested that no particular sub-population is responsible for communicating the aggressive traits to recipient cells and that all the EVs may act together to transfer the undesirable effects. Thus, for the next part of our study it was appropriate to try a number of compounds to establish if we could block the release of the heterogenous EVs population, rather than focussing on any single sub-population of EVs.

**Fig. 3:**
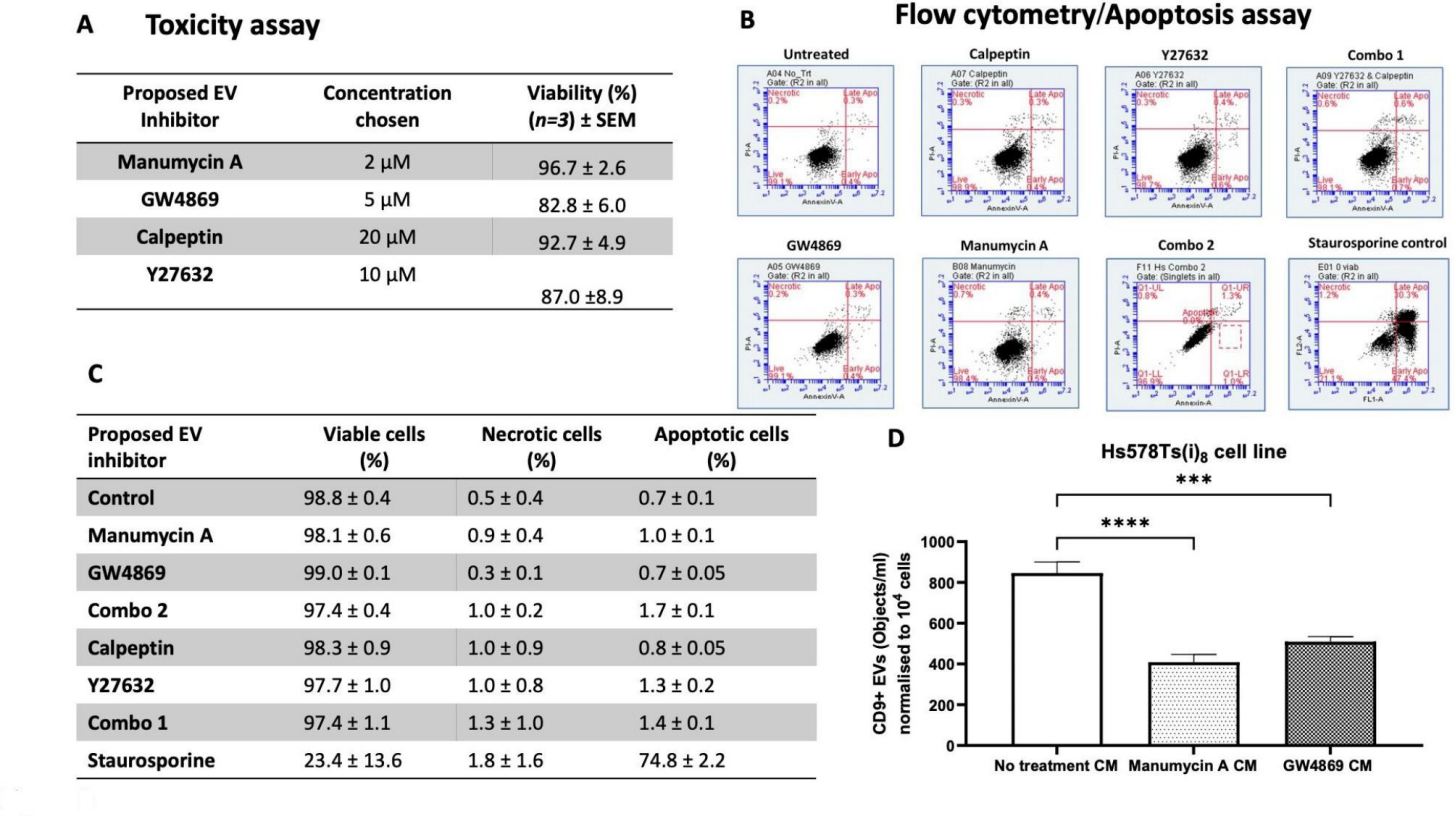
An acid phosphatase assay (**A**) was used to measure the viability of the Hs578Ts(i)_8_ cells after 48 h of inhibitor treatment. Representative dot plots are shown for annexin/PI flow cytometry (**B**) which was used to confirm toxicity at the chosen drug concentrations. Staurosporine (1 µM) was used as a positive inducer of apoptosis. Table (**B**) shows results from annexin V/PI flow cytometry. (**D**) A quick screening assay was developed in which cells were cultured in a 24-well plate and treated with drugs/compounds for 48 h. Afterwards, CM was collected and analysed for the presence of CD9 using imaging flow cytometry. CD9 + particles were detected and normalised to 1 × 10^4^ (**D**) Hs578Ts(i)_8_ cells. Tables show mean ± SEM of *n = 3* independent biological experiments. Graphs represent mean ± SEM of *n = 4* independent biological experiments. One-way ANOVA was used as statistical test. ***P < 0.001, ****P < 0.0001

To establish what compound concentrations to use to be able to determine if they were actually blocking EVs release rather than simply causing toxicity to the cells, acid phosphatase cytotoxicity assays were used on the Hs578Ts(i)_8_ cells. From this, the following concentrations were chosen: 2 µM manumycin A, 5 µM GW4869, 20 µM calpeptin and 10 µM Y27632 (Fig. 3(A)). Flow cytometry was used as an orthogonal method to confirm cell viability after treatment. Here the annexin V/propidium iodide (Annexin V/PI) assay was used to assess cell viability after treatment with each individual compounds alone or with the combination of calpeptin and Y27632 together (i.e. Combo 1) and the combination of GW4869 and manumycin A together (i.e. Combo 2) (Fig. 3 (B)). This analysis confirmed that the compounds did not result in toxicity at the concentrations we had we chosen based on the acid phosphatase assays (Fig. 3(C)).

Collecting EVs from solution, whether that be conditioned medium (CM) or another biofluid, is laborious. Thus, we performed a proof-of-concept analysis to establish if it might be possible to perform an initial “quick pre-screen” by directly analysing CM samples by FC to identify any possible changes in EVs quantities in solution in response to a compound. Manumcyin A and GW4869 were used for this investigation, with CD9 included as a representative EVs marker. This pre-screen suggested that manumcyin A and GW4869 significantly reduced the release of EVs (i.e., CD9 + EVs) (Fig. 3(D)).

### Decrease in EVs release by drug/compound treatment of cells

**Fig. 4:**
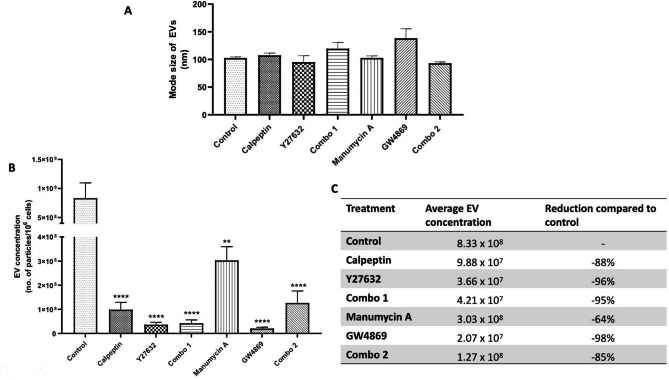
The (**A**) size and (**B**) numbers of released EVs were measured by NTA. The (**C**) % of release was calculated compared to the untreated control. Graphs (**A**) and (**B**) represent mean ± SEM of *n = 4* independent biological experiments. One-way ANOVA was used as statistical test. *P < 0.05, ****P < 0.0001

NTA analysis was performed to establish if any, or all, EVs might continue to be released following cell treatment with the compounds of interest. This analysis showed that some vesicles/particles were still detectable post-treatment, with no significant change in the size (nm) of the EVs released following treatment, although GW4869 apparently increased particle sizes a little when compared to the influence of other treatments and the control (Fig. 4(A)). However, as shown in Fig. 4(B), success in reducing EVs released was achieved with all treatments used including calpeptin, Y27632, Combo 1, manumycin A, GW4869 and Combo 2. While complete inhibition of EVs release did not occur, release of 64-98% of EVs was blocked, depending on the treatment used (Fig. 4(C)). Histograms illustrating EVs/particles’ concentrations and sizes are shown as Supplementary Fig. 1.

**Fig. 5:**
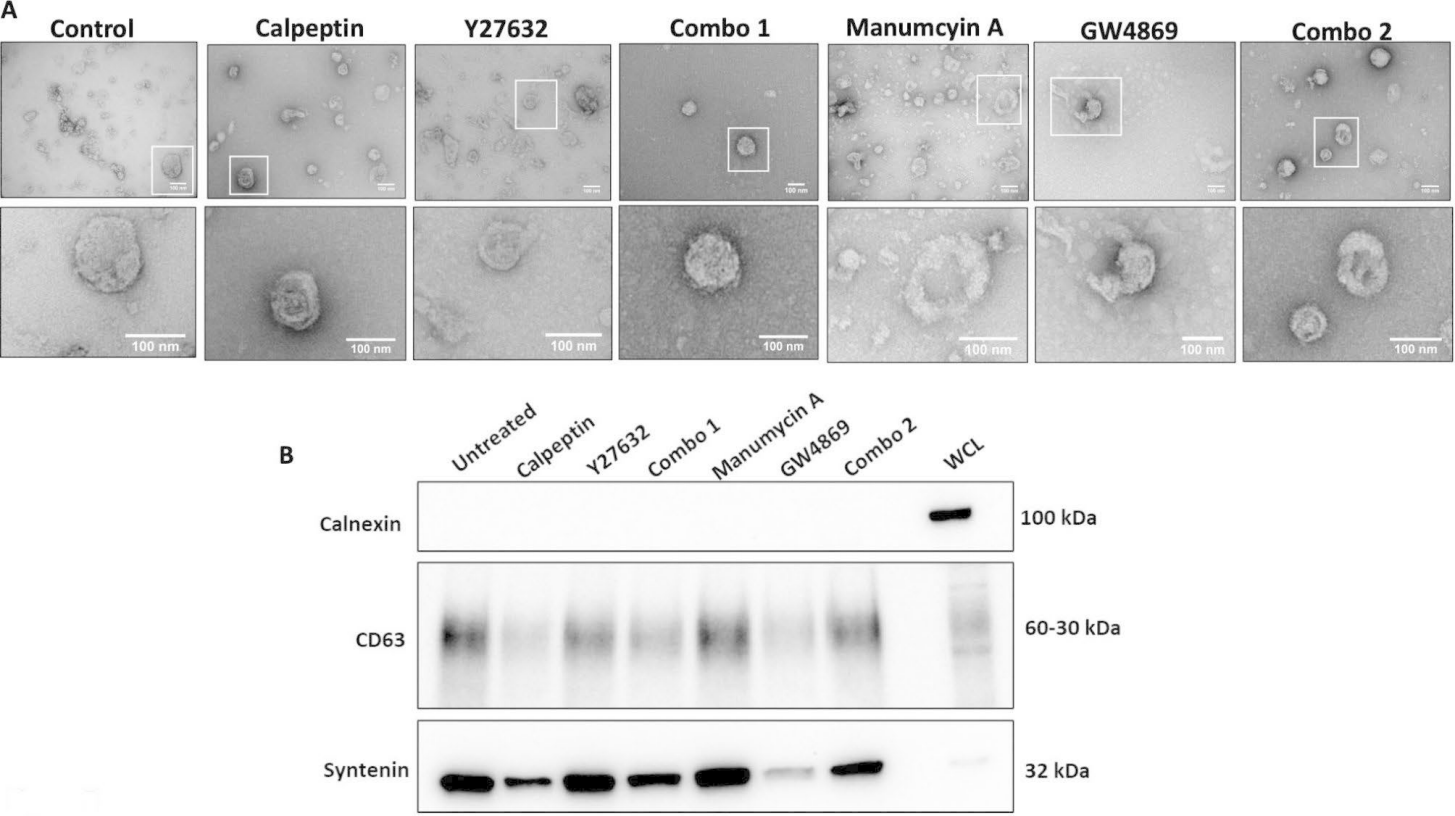
TEM analysis (**A**) of EVs. Images were taken at a magnification of 30000X. Scale bar = 100 nm. Immunoblot (**B**) analysis of EVs following treatment with compounds. Representative immunoblots are shown. Calnexin was used as a negative EV marker, and Hs578Ts(i)_8_ cell lysate was used as a positive control. WCL = whole cell lysate. Full-length blots/gels are presented in Supplementary Fig. 3

No method for EVs analysis is without some limitations and so MISEV2018 guidelines support the use of a number of methods for EVs characterisation. Thus, as well as analysis by NTA to establish changes in EVs/particle quantities, TEM and immunblotting were also performed. TEM showed rounded structures, typically of < 100 nm in size and representative of EVs in all samples (Fig. 5(A)). Like the control samples, the EVs that continued to be released post-treatment of the cells were typically heterogenous in size. Immunoblot analysis also showed that typical EVs’ markers were still detectable (here showing CD63 and syntenin as examples) and that the samples did not contain calnexin, which is sometimes considered a negative marker of EVs (Fig. 5(B)). Of note: as a fixed quantity of each sample needed to be loaded on the blots in all cases, we do not feel it is appropriate to compare quantities of markers detected. The immunoblotting analysis was performed rather to be able to determine presence or absence of EVs’ positive and negative markers.

### Functional analysis of EVs released after drug/compound treatment

**Fig. 6:**
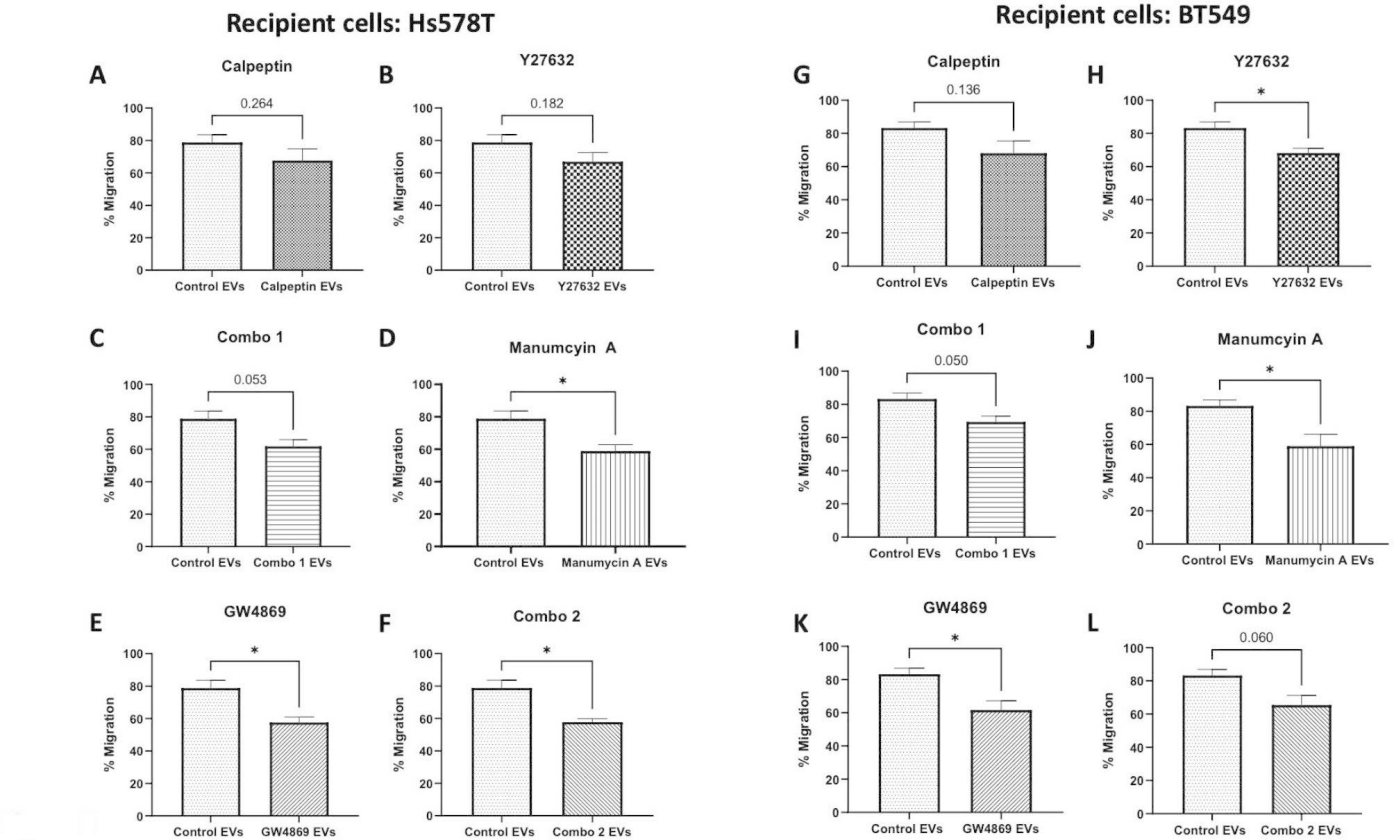
Hs578T (**A-F**) and BT549 (**G-L**) cell migration was monitored over 24 h after the addition of Hs578Ts(i)_8_-derived EVs following cell treatment with drugs/compounds. Control EVs are EVs released from untreated cells. Closure of the wound was measured using ImageJ. Amount of EVs added were dependent on the cell density for each recipient cell line. Graphs represent mean ± SEM of *n = 3* independent experiments. Student’s t-test was used as statistical test. *P < 0.05

Although we successfully blocked release of the majority of the EVs, we were also interested in investigating if the influences of EVs that continued to be released from the aggressive Hs578Ts(i)_8_ cells post-treatment would have changed. Thus, we evaluated migration of the parent/docile Hs578T cells and as a second recipient cell line, BT549. Of note, here the amount of EVs added accounted for the reduction of EVs’ release as measured by NTA, and when compared to untreated Hs578Ts(i)_8_ cells released EVs (i.e. control EVs). This study design was used to reflect how events might occur following blockage of most, but not all, EVs’ release. This analysis showed that the EVs that continued to be released after calpeptin (Fig. 6 (A)), Y27632 (Fig. 6 (B)) and Combo 1 (Fig. 6 (C)) did not have a significant effect on Hs578T migration. However, EVs that were released post-treatment with manumycin A (Fig. 6 (D)), GW4869 (Fig. 6 (E)) and Combo 2 (Fig. 6 (F)), compared to EVs from the control cells, caused significantly less migration of Hs578T cells. Similarly with BT549s, EVs released from calpeptin (Fig. 6 (G)), Combo 1 (Fig. 6 (I)), and Combo 2 (Fig. 6 (L)) treated cells did not have a substantial effect on BT549 migration. Treatment with EVs released after Y27632 (Fig. 6 (H)) manumycin A (Fig. 6 (J)) and GW4869 (Fig. 6(K)), compared to control EVs,induced significantly less BT549 migration. However, although all compounds tested blocked a high percentage (64–98%) of EVs release, following treatment with a number of the compounds or combinations there of, the low percentages of EVs that continued to be released were typically not remarkably less active at inducing migration. In fact, relatively speaking were often more active.

## Discussion

The heterogenous population of EVs released by TNBC cells can transmit the aggressive phenotype of their cells of origin to recipient cells. We and others have shown this transmission to occur to both normal and cancer cells. A logical way towards stopping these undesirable traits spreading would be to block release of the EVs involved in this communication. Thus, to determine specifically what EVs we would need to block, we first wanted to determine if the collective heterogenous population of EVs or a single sub-population of EVs was likely to be responsible [9].

The aggressive triple-negative breast cancer cell line variant Hs578Ts(i)_8_ releases a heterogeneous population of vesicles. For this purpose, the heterogenous EVs population and each of four EV sub-populations released from Hs578Ts(i)_8_ were collected using ultracentrifugation-based methods. Using methods for EVs characterisation that are aligned with MISEV2018 guidelines and that include NTA, immunoblotting, and TEM, we established that EVs were successfully collected. Using cancer cell migration (scratch/wound healing) as representative phenotypic assay, we confirmed that the heterogenous population of EVs induced significant migration of recipient cells. Furthermore, all 4 EVs sub-populations analysed also induced this effect. However, none more so than the heterogenous population. This suggested that all EV sub-populations are responsible, acting together, to transfer these phenotypic effects. Thus, to block transmission of such undesirable traits, efforts to block all heterogeneous EVs release from the cancer cells might be ideal.

Indeed, a number of compounds have been proposed to inhibit EVs release. Often those deductions were based on single studies of a single drug/compound and at concentrations at which the compound would be toxic to the cells, making it challenging to decipher inhibition of EVs release from cellular apoptosis and necrosis. Thus, we elected to evaluate the effects on EVs release of 6 compounds or combinations thereof, after first using orthogonal methods to establish their concentrations that would be non-toxic. Of note, the compounds that we selected for this purpose -based on our extensive review of this field [12]- were calpeptin, manumycin A, and Y27632 (that is reported to particularly affect EV trafficking) and GW4869 (that is reported to particularly affects lipid metabolism), as well as a combination of manumycin A and GW4869 and a combination of calpeptin and Y27632. Using this approach, we successfully established non-toxic concentrations of each.

To be truly confident of detecting any changes in EVs quantities released, we knew that we must collect released EVs, characterise, and quantify them. This is laborious. So, as a proof-of-principle, we were also interested in determining if we could perform a “quick screen”, using flow cytometry, of the medium conditioned by cells -targeting an accepted marker of EVs, CD9- to indicate if EVs’ release was blocked. This quick screen could then be validated (or otherwise) by the more laborious method. For this proof-of-principle purpose, we tested manumycin A and GW4869. Our results suggested that while a complete block of EVs release did not occur, a significant reduction in EVs release was achieved in response to both compounds.

Subsequently, a more thorough investigation was performed i.e. additional compounds were included and an advanced method of EVs collection, i.e. tangential flow filtration and density gradient, was used that allowed us to more comprehensively characterise EVs (by NTA, immunoblotting, and TEM), in line with MISEV2018 guidelines. Of note, this was something that, to the best of our knowledge, has not previously been done when performing studies of potential EVs inhibitors. Here we found that no compound and no combination of compounds completed blocked EVs release. However, we established that all 6 compounds/combinations tested very substantially (64–98%) and significantly inhibited EVs release at non-toxic concentrations. Moreover, the fact that the significant affects of manumycin A and GW4869 were predictable from our quick screen approach supports the potential relevance of employing this simpler approach as a starting point in future large-scale studies. Ultimately, based on our more comprehensive EVs collection and characterisation, GW4869 evidently had more influence on the EVs release (98% block) compared to manumycin A (64% block) than would have been predicted by the quick screen. Possible explanations for this difference between method outcomes include the fact that maybe CD9 + EVs were more affected by GW4869 than by manumycin A. Regardless, this phase of the study suggested that further development of our “quick screen” approach might be useful in the future and that all 6 compounds/combinations we tested successfully reduced EVs release from the TNBC cells.

It is noteworthy that, as mentioned above, other researchers have evaluated the influence of some of these compounds; *albeit* not on TNBC cells. Focussing on EVs from VSC 4.1 neurons post-GW4869 treatment, He et al. [18] measured protein quantities as a surrogate of EVs release, rather than using a method to specifically quantify EVs/particles. However, our results from protein analysis here indicated that total protein and EV quantities do not directly correlate with each other. This shows that protein analysis is not a reliable surrogate of EVs quantities, at least for EVs from TNBC cells. Rather, in our study TEM demonstrated the presence of some EVs with or without compound treatment of the donor cells. Furthermore, immunoblotting for positive and negative markers of EVs supported the TEM and NTA analysis. Interestingly, some studies replied on immunoblotting of protein only to claim changes in EV quantities. For example, Yue et al. [19]. reported that GW4869 inhibited EVs release from endothelial (HUVECs) cells, an observation based on a reduction in EVs markers (CD9 and ALIX) by immunoblots. Similarly, Faict et al. [20] used immunoblots for CD81 and TSG101 to conclude that GW4869 decreased EVs release from murine multiple myeloma 5TGM1 cells. Our results, in line with MISEV2018 guidelines, suggest that immunoblotting can help support the claim of EVs presence, but it alone cannot verify EVs presence or changes in their quantities. Arguably more comprehensive analyses -as we performed here- are required.

While, as detailed above, we very significantly reduced EVs release with all compounds, between 2 and 36% of the EVs (depending on the compound/combination) were not blocked and continued to be released. In this the first reported study that has also progressed to investigated the EVs that continue to be released after the use of potential inhibitors, we were interested in establishing if the phenotypic characteristics that these EVs transmit would have changed compared to those EVs released in the absence of any such treatment. Crucially, here we used EV quantities that reflected the EVs percentage reduction to, as much as possible, reflect what would be happening post-treatment. This was investigated with two TNBC recipient cell lines, Hs578T and BT549; again using migration (scratch/wound healing) assay as a representative assay. In all cases, with both recipient cell lines, we found that post-treatment EVs induced less -sometimes significantly less- migration of recipent cells than did EVs from untreated cells. The fact that this reduction in transfer of phenotype was somewhat modest, compared to the very substantial reduction in EVs release achieved, suggests that the small quantity of EVs that continue to be released post-treatment may be even more aggressive than formerly. It should be noted that the wound healing assay is a combination of proliferation and migration of cells. Mitomycin c treatment to prevent proliferation was not used here and, thus, is considered to be a limitation of how this assay was performed. To specifically evaluate migration in the future, cells treated with mitomycin c to prevent proliferation should be investigated.

## Conclusions

This study suggests that the combination of the heterogeneous population of EVs released from TNBC cancer cells, rather than a particular sub-population, is responsible for the transmission of undesirable phenotypic traits to recipient cells. Thus, preventing this transmission may require blocking release of all these EVs. A broad range of compounds and combinations thereof -at non-toxic concentrations- can signficantly reduce EVs release. Furthermore, the quick screen flow cytometry method that we reported here may be useful to help select drugs/compounds which change EVs release, prior to progressing to collection and comprehensive characterisation of the EVs. Post-treatment, despite the fact that only a small percentage of EVs continued to be released –as low as 2% in some cases– and so the ability of these EVs to transmit undesirable effects to recipient cells was reduced, this reduction was on a small scale relative to the substantial reduction in EVs release achieved. This suggests that, in response to EVs inhibitors, cancer cells may in fact release a small quantity of particularly potent EVs. Thus, while substantial success was achieved in blocking most EVs, effort to attain complete EVs block from cancer cells are warranted. Given that healthy cells also release EVs necessary for normal intercellular communication and so inhibiting their EVs release would be undesirable, innovative approaches to selectively deliver inhibitors to cancer cells (e.g. by their payloading to a cancer cell-targeted monoclonal antibody) may be a useful way to progress this research towards clinical benefit.

## Electronic supplementary material

Below is the link to the electronic supplementary material.

Supplementary Material 1

## Data Availability

While this manuscript does not include big data, all analysed and derivative raw data are available on request to Professor Lorraine O’Driscoll lodrisc@tcd.ie.

## List of abbreviations

ATCC: American Tissue Culture Collection
CM: Conditioned medium
EVs: Extracellular Vesicles
FBS: Foetal Bovine Serum
NTA: Nanoparticle Tracking Analysis
PI: Prodiuim Iodide
TEM: Transmission Electron Microscopy
