# Supplementary material for: An extensive study of potential inhibitors of extracellular vesicles release in triple-negative breast cancer
Source: BMC Cancer. 2023 Jul 13;23:654. doi: 10.1186/s12885-023-11160-2 (PMC10339474; doi:10.1186/s12885-023-11160-2)
Supplement: Supplementary file 1 — Supplementary Material 1 [file 12885_2023_11160_MOESM1_ESM.pdf]

Supplementary Figures

Suppl. Fig. 1

EVs released under Control Conditions

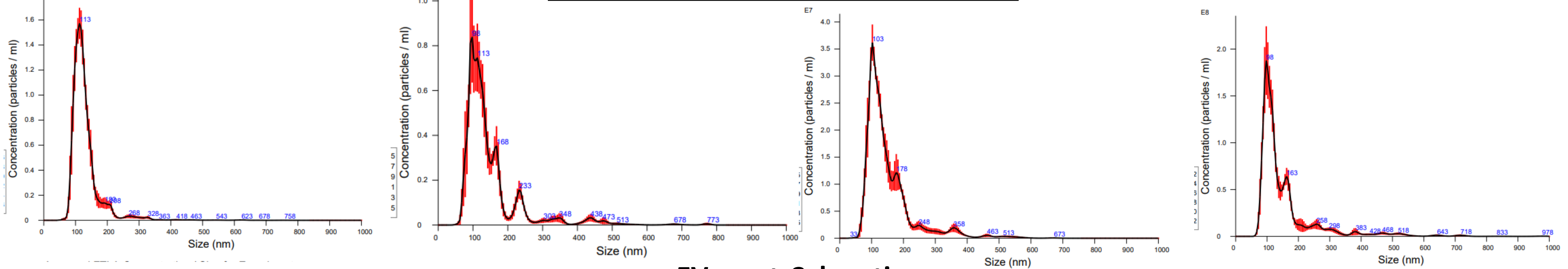

EVs post-Calpeptin

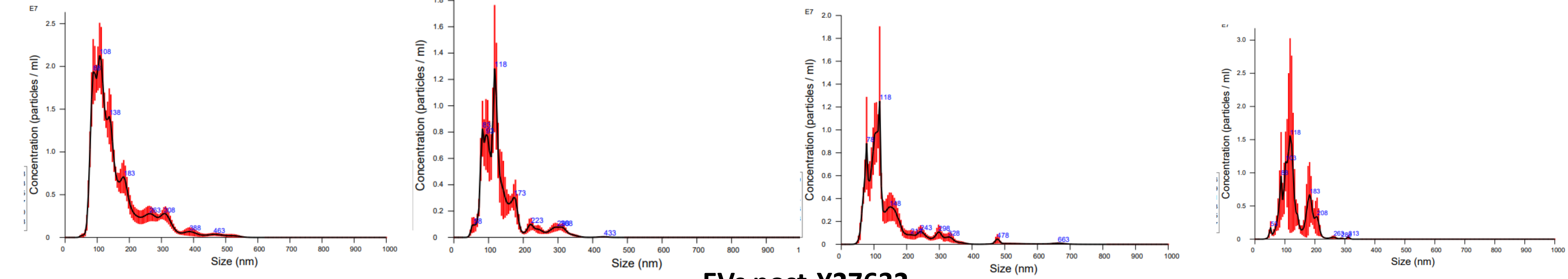

EVs post-Y27632

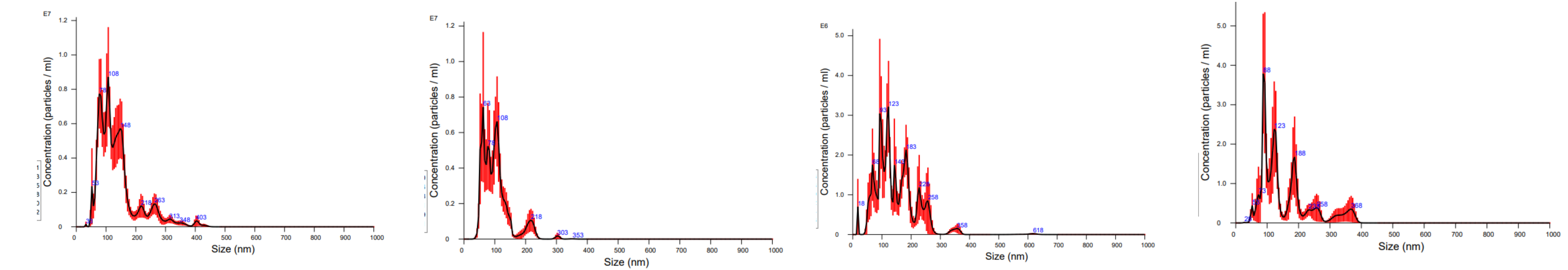

Suppl. Fig. 1 *(cont.)*

EVs post-Combo 1

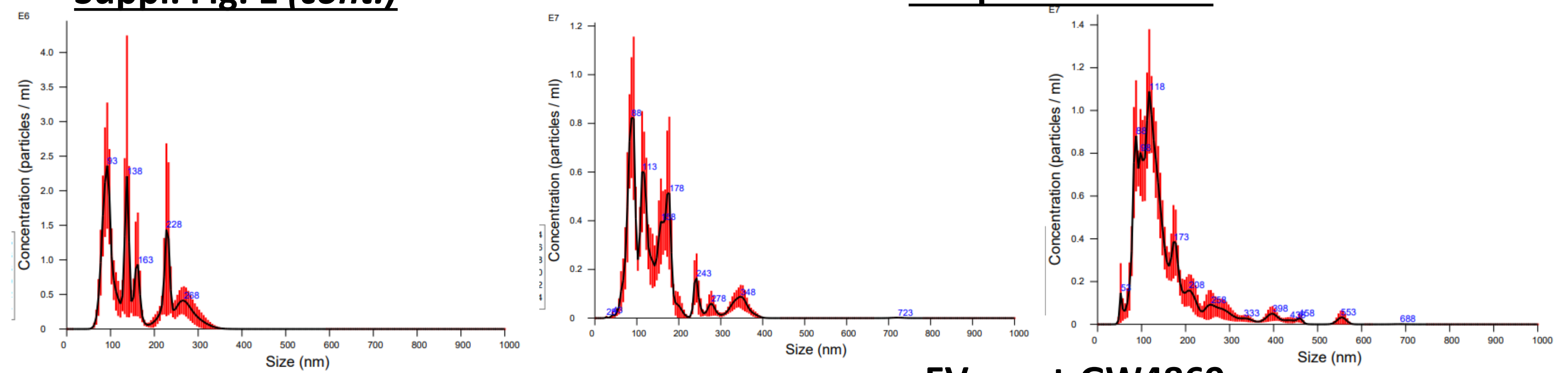

EVs post-GW4869

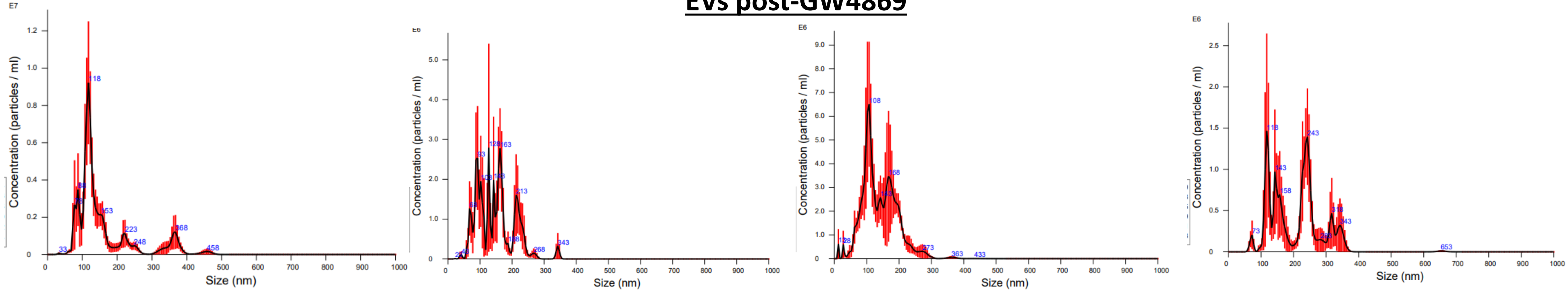

EVs post-Manumcyin A

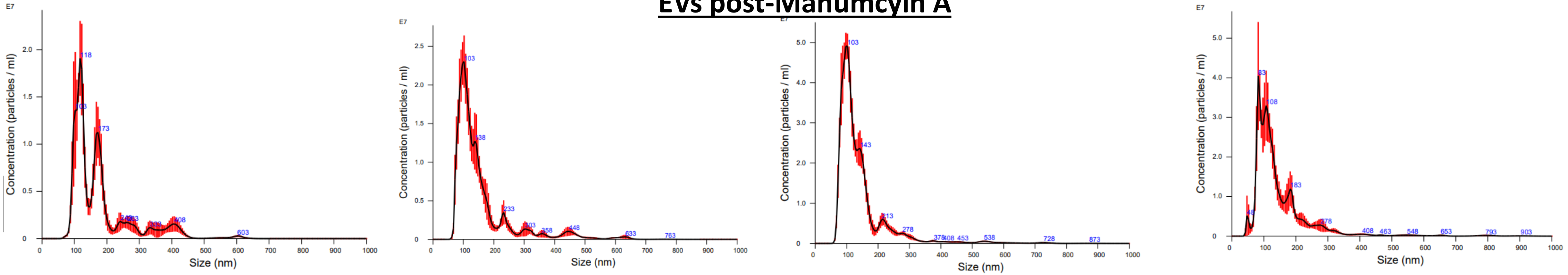

Suppl. Fig. 1 *(cont.)*

EVs post-Combo 2

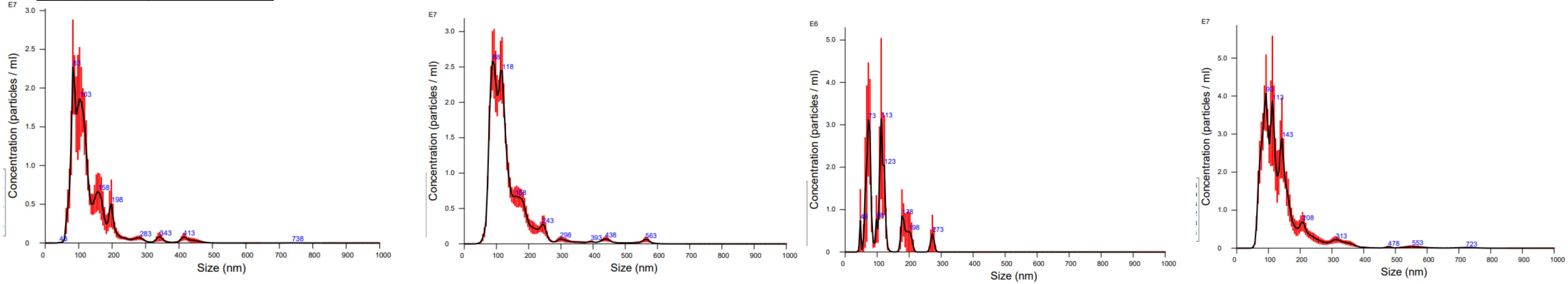

Supplemental Figure 1. Histograms illustrating EVs/particles concentrations and sizes

Suppl. Fig. 2

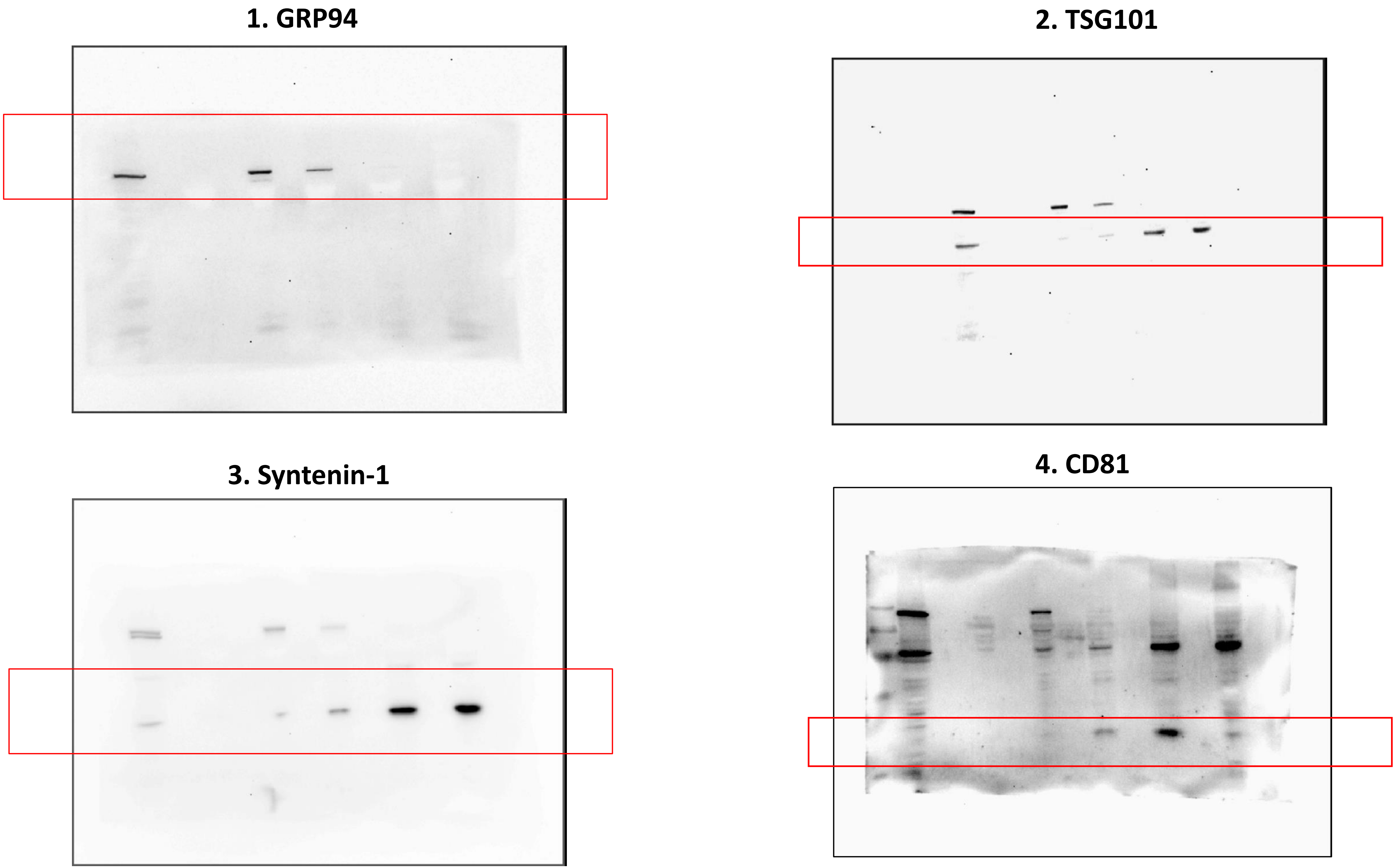

Supplemental Figure 2. Full length immunoblots (cropped versions in main manuscript)

Suppl. Fig. 3

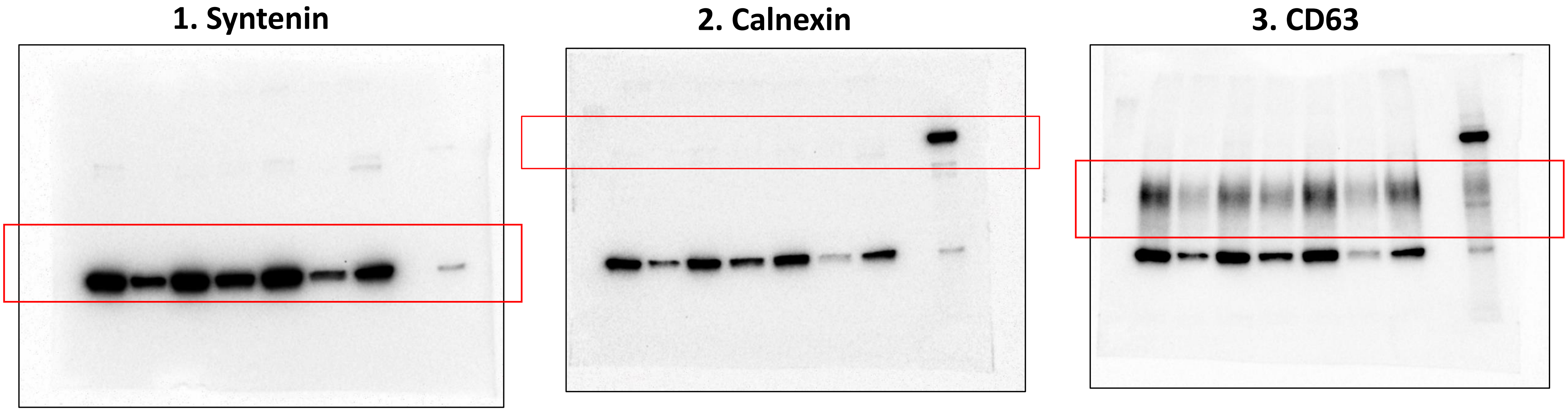

Supplemental Figure 3. Full length immunoblots (cropped versions in main manuscript)
